# “They are gaining experience; we are gaining extra hands”: a mixed methods study to assess healthcare worker perceptions of a novel strategy to strengthen human resources for HIV in South Africa

**DOI:** 10.1186/s12913-022-09020-z

**Published:** 2023-01-11

**Authors:** Deanna Tollefson, Nasiphi Ntombela, Sarah Reeves, Salome Charalambous, Gabrielle O’Malley, Geoffrey Setswe, Ann Duerr

**Affiliations:** 1 Department of Global Health, University of Washington, Seattle, USA; 2 The Aurum Institute, Implementation Research Division, Parktown, South Africa; 3 Youth Health Africa, Parktown, South Africa; 4 School of Public Health, University of the Witwatersrand, Johannesburg, South Africa; 5 Department of Health Studies, University of South Africa, Pretoria, South Africa; 6 Fred Hutchinson Cancer Research Center, Vaccine and Infectious Disease & Public Health Science Divisions, Seattle, USA

**Keywords:** Lay health workers, HIV/AIDS, Implementation science, Task shifting, Health systems strengthening

## Abstract

**Background:**

Lay health workers (LHWs) can support the HIV response by bridging gaps in human resources for health. Innovative strategies are needed to expand LHW programs in many low- and middle-income countries. Youth Health Africa (YHA) is a novel LHW approach implemented in South Africa that places young adults needing work experience in one-year non-clinical internships at health facilities to support HIV programs (e.g., as HIV testers, data clerks). While research suggests YHA can increase HIV service delivery, we need to understand healthcare worker perceptions to know if this is an acceptable and appropriate approach to strengthen human resources for health and healthcare delivery.

**Methods:**

We conducted a convergent mixed methods study to assess healthcare worker acceptance and perceived appropriateness of YHA as implemented in Gauteng and North West provinces, South Africa and identify issues promoting or hindering high acceptability and perceived appropriateness. To do this, we adapted the Johns Hopkins Measure of Acceptability and Appropriateness to survey healthcare workers who supervised interns, which we analyzed descriptively. In parallel, we interviewed frontline healthcare workers who worked alongside YHA interns and conducted an inductive, thematic analysis. We merged quantitative and qualitative results using the Theoretical Framework of Acceptability to understand what promotes or hinders high acceptance and appropriateness of YHA.

**Results:**

Sixty intern supervisors responded to the survey (91% response rate), reporting an average score of 3.5 for acceptability and 3.6 for appropriateness, on a four-point scale. Almost all 33 frontline healthcare workers interviewed reported the program to be highly acceptable and appropriate. Perceptions that YHA was mutually beneficial, easy to integrate into facilities, and helped facilities be more successful promoted a strong sense of acceptability/appropriateness amongst healthcare workers, but this was tempered by the burden of training interns and limited program communication. Overall, healthcare workers were drawn to the altruistic nature of YHA.

**Conclusion:**

Healthcare workers in South Africa believed YHA was an acceptable and appropriate LHW program to support HIV service delivery because its benefits outweighed its costs. This may be an effective, innovative approach to strengthen human resources for HIV services and the broader health sector.

**Supplementary Information:**

The online version contains supplementary material available at 10.1186/s12913-022-09020-z.

## Background

The global healthcare worker (HCW) shortage disproportionately impacts low- and middle-income countries (LMICs) [1]. This has been a particular challenge for the HIV response in LMICs, such as South Africa [2–5]. South Africa has the world’s largest HIV epidemic [6], which requires sufficient human resources for health to ensure HIV programs can be fully delivered [5, 7]. Insufficient human resources lead to excessive workloads for HCWs, which is known to reduce HCW motivation and contribute to attrition [8–10]. Use of lay health workers (LHWs), defined as those supporting health work without professional or paraprofessional qualifications, has long been advocated to increase human resources for health and thereby improve health care [11, 12]. LHWs are a recommended approach to support HIV service delivery [12, 13], as LHWs can assume HIV testing and counseling responsibilities to increase testing, treatment initiation, and retention in care [13–17]. Novel strategies are need to bolster the supply of LHWs, which remains insufficient in LMICs, including South Africa, due to funding constraints [18–21].

Youth Health Africa (YHA) is a novel LHW approach that places young adults needing work experience in one-year, non-clinical internships at health facilities to strengthen HIV service delivery. Like traditional LHWs, interns support clinics by task shifting and task sharing [22]. However, YHA is different from other LHW programs as it involves young adults working for only one year, when typically, LHWs in South Africa are older with longer assignments [20]. Moreover, YHA is a multisectoral program; while YHA seeks to strengthen the health system through LHWs, its primary goal is youth empowerment, providing interns work experience to increase youth employability and decrease unemployment [23].

Research suggests YHA may improve facility-based HIV service delivery [24], but HCW perspectives on this program are unknown. HCWs must perceive the YHA approach as acceptable and appropriate for it to be recommended as a LHW approach, as acceptability and appropriateness are paramount to successful program implementation [25, 26]. While HCWs are often appreciative of LHW programs because they result in task shifting and task sharing [27], HCWs can find LHW programs less acceptable or appropriate due to LHW incompetence or increased workplace competition [3, 11, 26, 28]. The novelty of the YHA approach means it is difficult to predict how HCWs perceive the program. We thus conducted a study to [1] describe the acceptability and appropriateness of YHA from the HCW perspective and [2] understand what promotes or hinders high acceptability and perceived appropriateness of the YHA approach.

## Methods

### Study design

We used a convergent mixed methods study design (QUAN + QUAL) to assess HCW perceptions of acceptability and appropriateness of YHA as implemented in South Africa. We defined acceptability as how satisfying or agreeable YHA was to HCWs and appropriateness as how well YHA fit HCW/facility needs [25]. We concurrently collected quantitative data through surveys and qualitative data through interviews using a multilevel sample of HCWs. We analyzed data separately, then merged findings for interpretation. We used a mixed methods approach for purposes of complementarity and triangulation, which fostered a more comprehensive understanding of HCW perceptions than either method would have alone [29].

### YHA program

YHA collaborates with PEPFAR implementing partners who support clinics to place interns in facilities that need additional human resources to support HIV service delivery. YHA interns are 18–34 years old with secondary education but no employment experience. They are assigned to be program interns (e.g., HIV testers, peer navigators) or administrative interns (e.g., data entry, filing clerks). YHA leads a broad 3–5-day training for interns, with health facilities providing technical training as needed. YHA pays interns stipends and provides monthly professional development trainings. Interns work alongside HCWs at facilities and are formally supervised by a HCW working for the PEPFAR implementing partner. YHA is modeled after internship programs implemented at South African businesses, where private organizations fund internships, incentivized by the country’s Broad-Based Black Economic Empowerment (B-BBEE) policy [30].

### Study population

The study population comprised HCWs who supervised or worked with YHA interns, which we refer to as “intern supervisors” and “frontline HCWs”, respectively (Supplement 1). Intern supervisors were tasked by the PEFPAR implementing partner to provide supportive supervision to interns and often worked across many facilities. Frontline HCWs were staff who worked alongside interns (co-workers) and the nurses who helped manage/lead the HIV unit (facility managers).

We conducted surveys with intern supervisors because they offered a broad perspective on YHA, having engaged with multiple interns across many facilities. We interviewed frontline HCWs to gain in-depth understanding of their experiences working with interns and perceptions of program benefits.

### Setting

While YHA has been implemented across South Africa, this study was conducted among HCWs at facilities in Gauteng and North West (NW) provinces where Aurum Institute is PEPFAR implementing partner. Surveys were completed by intern supervisors working across these provinces. Interviews were conducted in facilities from two districts: Ekurhuleni (Gauteng) and Ngaka Modiri Molema (NW).

### Sampling

All intern supervisors supporting YHA were invited to take the survey. Concurrently, a sample of frontline HCWs were selected for interviews using a two-step process. First, we selected facilities using random sampling, stratifying by geography (NW versus Gauteng) and facility type (clinic versus community health centers [CHCs]/hospitals), as we hypothesized that experience with YHA may vary based on these elements. Five clinics and three CHCs/hospitals were selected from each region. We then used purposive sampling to select frontline HCWs to interview. At each facility, we selected an available facility manager and a co-worker who worked closely with a YHA intern.

### Data collection

From March–May 2021, intern supervisors completed a self-administered, electronic survey, which included close-ended questions: 15 to measure acceptability, 15 to measure appropriateness, and others on supervisor role, supervisor characteristics, and program implementation. The questions on acceptability and appropriateness were adapted from the Johns Hopkins Dissemination and Implementation Science (JHD&I) Measure of Acceptability and Appropriateness [31], which was validated and used in other LMICs [32–36]. These questions were answered on a four-point scale (1 = “Not at all,” 2 = “A little”, 3 = “A moderate amount,” or 4 = “A lot”). We made minor adaptions to fit YHA and South African contexts and piloted the survey before use. The survey was in English and implemented using REDCap electronic data capture tools, which were hosted at the Institute of Translational Health Sciences.

During the same period, we conducted semi-structured in-person interviews with frontline HCWs, which included questions on their experience with YHA and benefits and challenges of the program. Two questions asked about acceptability and appropriateness, pulled from the JHD&I measures [31]. Research assistants from Aurum Institute conducted interviews in English or local languages (i.e., Setswana in North West and IsiZulu or Sesotho in Gauteng), based on interviewee preference. Interviews lasted between 30–60 min. All interviews were recorded, transcribed and translated into English.

### Quantitative analysis

We calculated a total and average score for acceptability and appropriateness for each respondent. When responses were missing or categorized as “I don’t know”, we weighted final scores based on existing responses. We then calculated the mean and standard deviation for acceptability and appropriateness in Stata 15 (StataCorp LLC., College Station, TX). We graphically summarized responses for each question comprising the acceptability and appropriateness measures in R 3.6.1 (R Core Team 2019, Vienna, Austria), using *ggplot2* and *HH* packages [37, 38]; missing responses or those responding “I don’t know” were excluded. Finally, we conducted bivariate logistic regression analyses in R to explore whether intern supervisor characteristics or their roles were associated with acceptability and appropriateness.

### Qualitative analysis

We used Dedoose 9.0.17 (SocioCultural Research Consultants LLC., Los Angeles, CA) to conduct an inductive, thematic analysis. A primary coder developed a codebook based on preliminary review of transcripts. The primary and secondary coder coded three transcripts independently and came to consensus on code applications and an expanded codebook. The primary coder coded all transcripts and further refined the codebook. The secondary coder reviewed all coded transcripts and the updated codebook. Both came to consensus on all application of codes and collaboratively identified themes emerging from the interviews.

### Integration

**Fig. 1:**
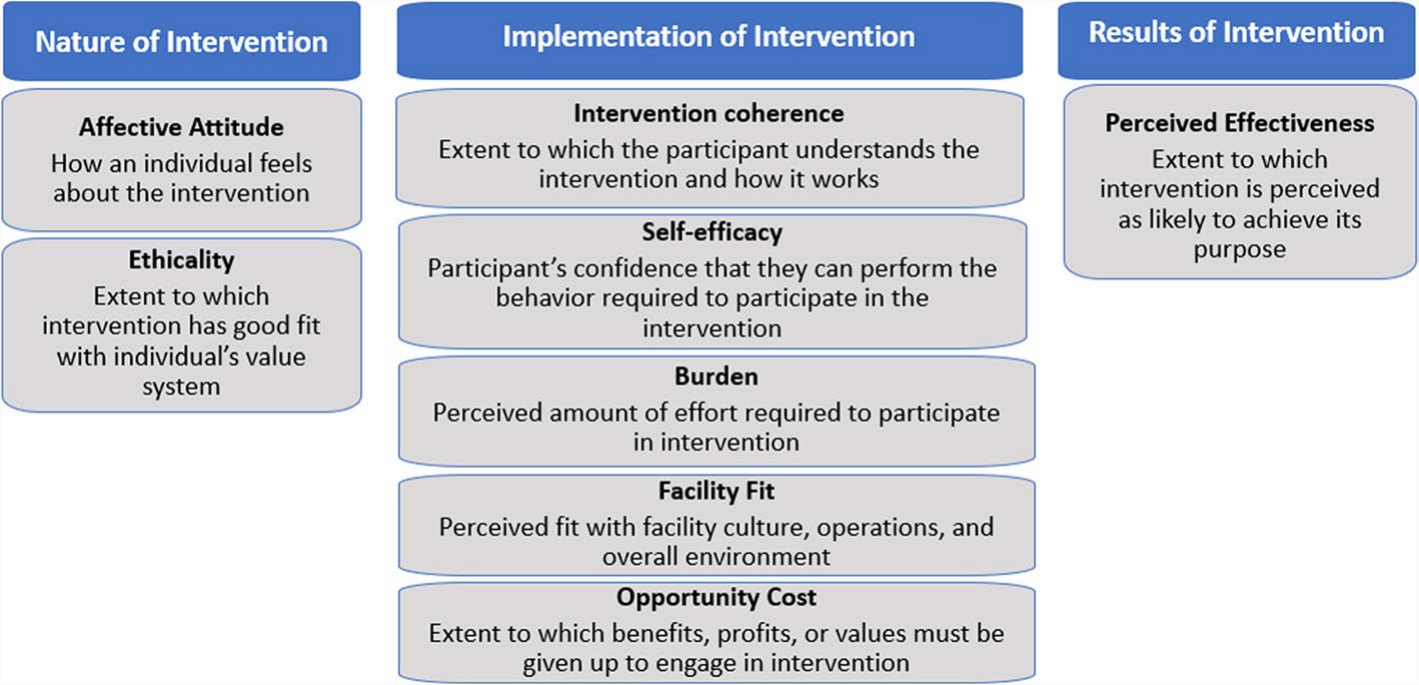
Overview of the expanded Theoretical Framework of Acceptability (TFA) domains used in mixed methods analysis. Original domains are as defined by Sekhon, et al. [39], with addition of one domain, ‘facility fit’, influenced by the definition of ‘appropriate’ by Proctor, et al. [25]. Domains were grouped into three groups by this study’s authors

We compared quantitative and qualitative findings and interpreted results together to present a holistic view of acceptability and appropriateness from the HCW perspective. To facilitate integration, we matched survey questions and interview themes to the domains of the Theoretical Framework of Acceptability, which was created to facilitate an understanding of what impacts acceptability of healthcare interventions [39]. While this framework’s definition of acceptability is inclusive of appropriateness, we added a domain, ‘*facility fit’*, to better fit Proctor’s definition of appropriateness [25]. To further support interpretation of our data, we divided this framework’s domains into three naturally occurring groups: nature of the intervention, implementation of the intervention, and results of the intervention. The domains of this framework are outlined and defined in Fig. 1.

### Ethics, consent, and permissions

This study was approved by University of Witwatersrand (Johannesburg, South Africa) and University of Washington (Seattle, USA), along with provincial and district-level ethics committees where it was conducted. Participants provided written consent before engaging in surveys or interviews.

## Results

### Participants

Sixty of 66 eligible intern supervisors completed surveys (91% response rate) (Table 1). Altogether, they supervised 364 interns at 180 facilities. Almost half had served as intern supervisor for at least one year, giving them experience with more than one intern cycle. We also interviewed 33 healthcare workers (16 facility managers, 17 co-workers) from 16 facilities (Table 2). Over half of facilities hosted 2–3 interns at the time when interviewed. Almost half of interviewees had worked at their facility for at least three years.

**Table 1:** Characteristics of survey respondents (*n* = 60)

**Survey Respondent**^**a**^	**n**	**%**
**Location**		
Northwest	33	55%
Ekurhuleni	27	45%
**Current Position**		
Data Monitor	18	30%
Counselling Supervisor	17	28%
Retention Supervisor	17	28%
Professional Nurse	1	2%
Data Capturer	1	2%
Other	6	10%
**Years in Current Position**		
Less than 1 year	9	15%
1–2 years	22	37%
3–5 years	26	43%
6–10 years	2	3%
11–15 years	1	2%
**Duration as intern supervisor**		
< 6 months	17	28%
6 months to 1 year	16	27%
≥ 1 year	27	45%
**Number of facilities where they supervise interns**		
1	17	28%
2	15	25%
3 to 4	13	22%
5 to 9	8	13%
10 or more	4	7%
None currently	3	5%
**Number of Interns they currently supervise**		
1	12	20%
2 to 3	16	27%
4 to 5	10	17%
6 to 10	2	3%
More than 10	7	12%
None currently	3	5%
**Roles of interns they supervise**^**b**^		
Programmatic	39	65%
Administrative	28	47%

^a^Surveys were conducted amongst healthcare workers who served as intern supervisors for Youth Health Africa

^b^Facilities could have both programmatic and administrative interns

**Table 2:** Characteristics of interviewees (*n* = 33) and their facilities (*n* = 16)

	**n**	**%**
**INTERVIEWEES**^**a**^	**33**	**100%**
**Facility Type**		
Clinic	17	52%
CHC/Hospital	16	48%
**Interviewee Position**		
Nurse/Clinician	18	55%
Admin Clerk	6	18%
Testing Counsellor	5	15%
Data Capturer	3	9%
Tracer	1	3%
**Number of Years at Facility**		
≤ 1 year	4	12%
2–4 years	8	24%
5–9 years	12	36%
≥ 10 years	8	24%
*Missing*	*1*	*3%*
**FACILITIES**	**16**	**100%**
**Number of Interns at facility**		
1	3	19%
2 to 3	9	56%
4 to 5	0	0%
6 to 10	1	6%
10 +	2	13%
*Missing*	*1*	*6%*
**Roles of interns at facility**		
Admin	12	75%
HTC	8	50%
Tracer	6	38%
Other	3	19%
*Missing*	*1*	*6%*

^a^Interviewees were healthcare workers who worked alongside Youth Health Arica interns

### Quantitative findings

**Fig. 2:**
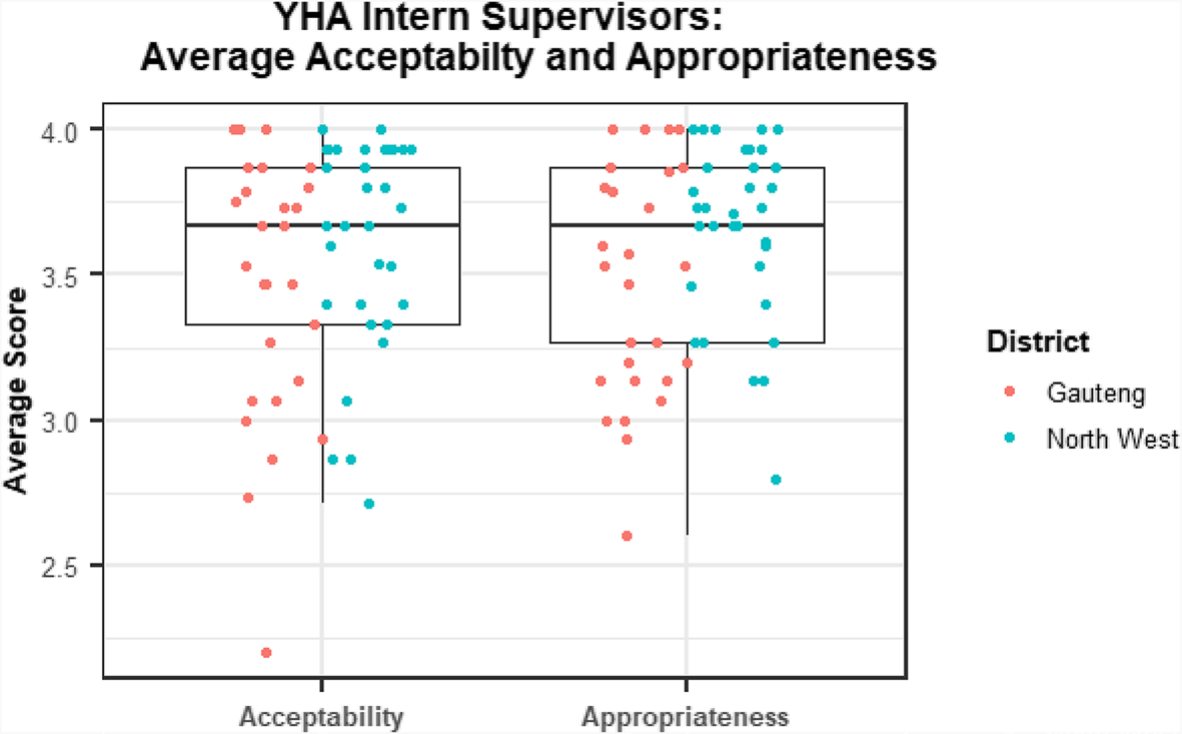
Distribution of overall average acceptability and appropriateness scores for intern supervisors, by province (*n* = 60). Average scores of acceptability and appropriateness correspond to the following scale: 1 = Not at all, 2 = A little bit, 3 = A moderate amount, or 4 = A lot

Surveys found YHA was acceptable and appropriate to intern supervisors. On a four-point scale, the average score for acceptability was 3.5 (standard deviation: 0.4), while the average score for appropriateness was 3.6 (0.4). Fifty-three respondents (89%) had average scores that rated the program at least moderately acceptable (≥ 3.0), and 57 (95%) had average scores that rated the program at least moderately appropriate (≥ 3.0) (Fig. 2). There were no associations with respondent characteristics (gender, profession, years in current position) or their roles with YHA (duration as supervisor, number of interns supervised, number of facilities requiring supervision) and acceptability or appropriateness (Supplement 2). Intern supervisors in Gauteng and those supervising only administrative interns had lower perceptions of acceptability and appropriateness, but differences were not significant.

**Fig. 3:**
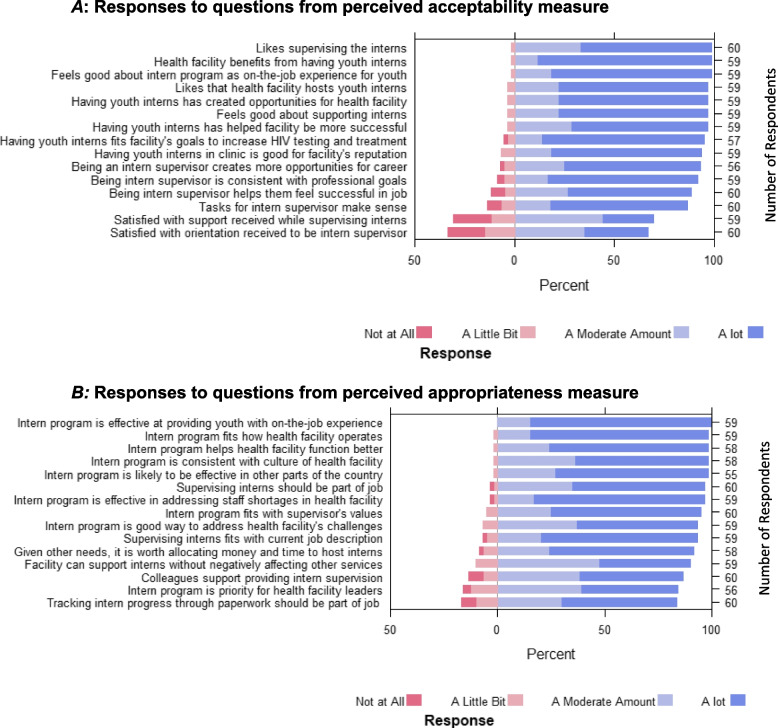
Summary of responses to acceptability and appropriateness questions on survey (*n* = 60). *A*: Responses to questions from perceived acceptability measure. *B:* Responses to questions from perceived appropriateness measure

While acceptability and appropriateness measures were highly scored overall, there was a range in positive responses for individual questions (Fig. 3). For the acceptability measure, top scoring questions were related to the nature of the program and its results, while top scoring questions for the appropriateness measure were related to program results and its general fit with the health facility. For both measures, the lowest scoring questions were related to the clarity and burden of the intervention and competing priorities. Two questions related to satisfaction with program orientation and support provided for supervision scored much lower than other questions (Fig. 3A).

### Qualitative findings

Most frontline HCWs reported that YHA was highly acceptable and appropriate. No respondents reported low levels of satisfaction or fit with YHA. Qualitatively, we observed no patterns in perceptions of acceptability or appropriateness by HCW role (facility managers versus co-workers), facility type, or geography. One nurse’s words capture the majority opinion:

*“There are no disadvantages. I have been singing praises thus far, that they [the interns] have helped us a lot.” (NW-14, Clinic)*

We identified four major themes that explained HCW perceptions on acceptability and appropriateness of YHA (Table 3).

**Table 3:** Key themes from interviews with frontline healthcare workers (HCWs) (*n* = 33) with supporting quotations

Theme	Summary	Illustrating Quotes
**Theme 1: YHA is mutually beneficial to HCWs and interns**	HCWs express strong affinity for YHA because they find interns to be a useful set of extra hands to support a HCW’s workload and they believe YHA benefits youth and broader society	*“*They are gaining experience; we are gaining extra hands. We are benefiting; they are benefiting.” *(NW-6, Nurse, CHC/Hospital)* “[The interns] are so helpful, helping hands. Whatever they do.” *(NW-5, Data capturer, CHC/Hospital)* “I feel so happy because…you grow a young kid who knew nothing, and you start seeing how much impact you play for her to be here.” (*NW-11, Counsellor, Clinic)*
**Theme 2: Facilities need agency when implementing YHA**	HCWs appreciated having a say in how YHA was implemented at their facility and became frustrated if they lacked this agency. Lack of agency was connected to lack of communication with YHA	“The intern program was not properly introduced to us….The first time I saw and heard of this program–the first time–I came in the morning, I found…they were navigators there….. So now, it was causing a confusion in the sense that you don't know who are these people, they were not introduced….” *(Gauteng-10, Nurse, CHC/Hospital)* “It seems as if now I am manager only [in a] vacuum because…when they take [the YHA intern] for the meeting they [do] not inform me….She doesn’t report to me, even now she is not here, I don’t know where is she.” *(NW-10, Nurse, Clinic)*
**Theme 3: Short internships can be burdensome**	The short nature of an internship can be burdensome, as HCWs spend time training interns and grow dependent on their support for them to leave after one year with no guarantee of replacement	“Honestly, the disadvantage [of YHA]? They employ people who don't have any experience. I have to come and show them the ropes, how are we doing the work here….For the first two weeks it was difficult, but my colleague, I think she is adapting very fast. So for now I don't have a problem, everything works smoothly.” (*NW-16, Admin Clerk, Clinic)* “If they are leaving or [the] internships ends, there will be a gap, where I would be alone again, lots of work, with backlog.” (*NW-5*, *Data capturer, CHC/Hospital)*
**Theme 4: Success from YHA is more than numbers**	HCWs believed YHA led to success in numerous ways in addition to progress towards HIV targets, such as smoother facility operations, improved HCW morale, and improved patient care	“[YHA] benefits patients in a lot of ways, like for instance, our waiting time is reduced, our nursing care has improved, and our patient survey says a lot.” *(Gauteng-13, Nurse, CHC/Hospital)* “You see, really if you have too much workload, you end up being exhausted and exhaustion brings burnout and end up with much absenteeism, so if we have more people working together, everything goes smoothly.” *(NW-17, Nurse, Clinic)* “But now, it is easy when they are here. The work is…going smooth. The files, the doctors when they come, they find that the files are already on the chairs. They are ready because they are here since the morning. They prepare the files the day before….They put them on the boxes…..And when the patients come, they don't go far away, the files are here, nearer, they just take the file, then they take the file to the nurses, they do the vital signs, then the patients go to the doctor. It is very easy. Working is easy, smoothly.” *(Gauteng-16, Nurse, CHC/Hospital)*

#### Theme 1. YHA is mutually beneficial to HCWs and interns

Frontline HCWs expressed strong affinity for YHA because they viewed it to be mutually beneficial, benefiting HCWs by being “extra hands” to do work at facilities and benefiting interns and broader society by providing youth with employment and job training. This theme is a synthesis of two key ideas present in interviews: that interns reduced HCW burdens and that HCWs felt good helping interns.

HCWs strongly emphasized that they were overworked as facilities were understaffed. They reported that interns alleviated some of this burden by offloading work through task sharing with non-clinical staff and task shifting with nurses.

*“Knowing that every day there is a person who is going to help with the simple tasks reduces the workload so that we can focus more on the clients.” (Gauteng-4, Nurse, Clinic)*

In describing how YHA reduced facility workload, HCWs emphasized the interns’ professionalism. HCWs frequently noted that interns were young and lacked experience, but they described interns positively (e.g., respectful, hardworking, committed, and amiable). While interns did need initial training and occasional coaching on professionalism, they were ultimately viewed as competent, independent workers who took initiative to accomplish tasks.

*“They are able to do everything by themselves, without us asking them, "Did you do this? Did you do that?" They know exactly what to do.” (NW-6, Nurse, CHC/Hospital).*

While HCWs liked the YHA program because it helped them, it was common for HCWs to say the first reason they liked the program was because it benefited interns. HCWs described appreciating that YHA provided immediate support to youth (e.g., wages to support their families), how it empowered youth to have better futures, and how it would better society by improving the skills of South Africa’s workforce. Co-workers often described liking the program because it provided immediate relief to a youth’s challenging economic circumstances, while facility managers, who tended to be older, found satisfaction in mentoring youth, as they believed this mentorship would improve the youth’s, and society’s, future*.*

*“I like [YHA] because at least they give these kids a chance of working. You know, some of them are coming… from schools, some of them are mothers, which they don’t have a job, they need a job you know.” (Gauteng-9, Data capturer, Clinic).*

*“[YHA] has also given me the satisfaction as a human being….that at least I am contributing to this, to our [youth]…., who will be our future tomorrow.” (Gauteng-13, Nurse, CHC/Hospital)*

#### Theme 2. Facilities need agency when implementing YHA

While HCWs were generally enthusiastic about YHA, they expressed frustration about being excluded from the planning process. HCWs described feeling unprepared for interns and felt interns were sometimes assigned to roles that did not match facility needs because HCWs were not consulted.

*“We only got clerks….Our challenge here is we don’t have data capturers…I wish they could have brought more data capturers and counselors.” (NW-3, Nurse, Clinic)*

However, once interns were placed at clinics and HCWs assumed responsibility for the interns, they integrated easily into clinic operations.

*“They have blended simply, and they have managed to just come to the facility and become part and parcel of us and became like a family.” (NW-14, Nurse, Clinic)*

Having this agency promoted the sense that YHA fit the facility, which fostered a high affinity for the program among most HCWs. Facility managers experiencing the poorest communication with YHA were apt to feel they lacked agency in directing the intern’s work. Though outliers, these HCWs expressed less satisfaction with the program.

#### Theme 3: Short internships can be burdensome

HCWs explained that while interns were helpful, the short internship period could be burdensome. HCWs were required to provide interns on-the-job training, which was time consuming, specifically for co-workers working closest with interns. Many HCWs were concerned about this upfront investment as interns would be present for only one year. Moreover, HCWs expressed concern that they had come to depend on interns, who were on time-limited contracts. HCWs frequently described wanting to lengthen internships or find a way to permanently hire interns to reduce this burden.

*“It is difficult when you have people you have trained then they have to go, then you have to start again. And we are not getting any remuneration for that, so it is tiring.” (Gauteng-12, Nurse, Clinic)*

#### Theme 4: Success from YHA is found in more than numbers

While frontline HCWs believed YHA helped them reach HIV targets, they appreciated YHA because it brought success in other ways. HCWs described how interns created a “smoother” working environment, such as improving patient flow by expediting retrieval of patient files. HCWs also described how YHA improved morale, as it reduced workload and ensured staff could step away without worry of interruption to services.

*“I feel better because I am no longer overloaded with work. Even if I am sick, I know there is a back-up then. The [YHA] data capturer will come and work. Everything will be well. (Gauteng-11, Data Capturer, CHC/Hospital)*

HCWs also believed YHA benefited patient care, especially in terms of improved services for young people. Many HCWs explained their struggles to test young people for HIV, due to limited hours for HIV testing and counseling and large age differences between counselors and adolescents. HCWs felt YHA interns bridged these gaps by enabling facilities to provide testing for extended hours with younger counselors.

### Integrated findings

Both intern supervisors and frontline HCWs found the YHA approach to be highly acceptable and appropriate. When we integrated findings from these two groups using the Theoretical Framework of Acceptability (TFA), we saw that intern supervisors and HCWs expressed similar sentiments about the YHA approach (i.e., the quantitative and qualitative findings converged), even though these HCWs had different roles in the program. This enabled us to identify elements of the program and its implementation that promoted or hindered acceptability and appropriateness of YHA among HCWs, which we discuss by TFA domain (Table 4).

**Table 4:** Joint display of factors promoting or hindering acceptability and appropriateness of Youth Health Africa

TFA^a^ Domain	Facilitator/ Barrier	Integrated Findings	Key Data	
			**Interviews**	**Surveys**^**b**^
**Nature of YHA**				
Affective Attitude	Facilitator	HCWs liked participating in YHA, specifically because interns addressed healthcare worker shortages	Theme 1: YHA is mutually beneficial (*Interns alleviate HCW burdens*)	Almost everyone liked supervising interns (98%) and having facilities host interns (97%)
Ethicality	Facilitator	HCWs liked that participation in YHA gave them chance to support young people and improve future society	Theme 1: YHA is mutually beneficial (*HCWs appreciate helping interns*)	Almost all felt good about the program as an on-the-job experience for youth (98%) and felt good about supporting the interns (97%)
**Implementation of YHA**				
Intervention Coherence	Barrier	HCWs lacked fully understanding of YHA and what was expected of them, needing more communication with YHA staff in early stages of program	Theme 2: Facilities need agency (*Poor communication*)	Many felt tasks they were asked to do as an intern supervisor made sense (87%); fewer (67%) were satisfied with the orientation they received to be supervisor
Self-Efficacy	Facilitator	HCWs were well-equipped to manage and supervise interns because this was similar to overseeing other staff	Theme 2: Facilities need agency (*Easy integration)*	Most thought supervising interns aligns with their job description (93%); almost all had prior supervisory experience (98%)
Facility Fit^a^	Facilitator	Youth interns were integrated easily into facilities because HCWs assumed responsibility for them once at facilities	Theme 2: Facilities need agency (*Easy integration*)	Almost all felt the intern program fit with the health facility culture (98%) and how the health facility operated (98%)
Burden	Barrier	Supporting YHA can be burdensome, as frontline HCWs had to train interns and interns added to supervisory responsibilities for intern supervisors	Theme 3: Short internships can be burdensome	Many HCWs viewed tracking intern progress as something they should do in their job (83%); fewer were satisfied with the support received while supervising interns (69%)
Opportunity Cost	Mixed	Opportunity costs appear low for all HCWs. While frontline HCWs think current benefits of program exceed costs, they express reservations that costs could exceed benefits if there are no plans to sustain help from interns	Theme 3: Short internships can be burdensome	Most felt health facilities could support interns without negatively affecting other services (90%) and that it was worth allocating resources to host interns at health facilities (91%). 85% thought benefits of YHA were equal to/outweighed time required of HCWs
**Results of YHA**				
Perceived Effectiveness	Facilitator	HCWs perceive YHA to be effective, contributing to a wide range of positive outcomes for healthcare workers, the facility, and its patients	Theme 1: YHA is mutually beneficial Theme 4: Success is more than numbers	Almost everyone felt health facilities benefited from having youth interns (98%), that YHA was effective in addressing staff shortages (96%), and that youth interns in clinics helped facilities be more successful (96%)

^a^The Theoretical Framework of Acceptability (TFA) was developed by Sekhon et al. 2017 [39] ‘Facility fit’ was added by study authors

^b^ All responses are presented for scores of ‘moderate’ or ‘a lot’

HCWs genuinely liked YHA as they found it provided the facilities with much-needed support (TFA domain: *affective attitude*) and they agreed with its higher goal of empowering youth through the internships (*ethicality*). Other elements that promoted acceptability/appropriateness of the program included HCWs finding it easy to integrate interns into their jobs (*self-efficacy*), the program aligning with facility culture (*facility fit*), and HCW belief that interns helped facilities be more successful (*perceived effectiveness*).

Elements hindering acceptability and appropriateness included having an inadequate introduction to or communication with the program (*intervention coherence*) and the time required to train, and to a lesser extent, supervise interns (*burden)*. We noticed divergence in whether HCWs perceived the *opportunity cost* of YHA interns to promote or hinder acceptability/appropriateness; while the benefits of YHA were widely believed to outweigh costs, frontline HCWs worried this balance could reverse if there was not a plan to ensure a sustained intern presence at facilities.

## Discussion

In this study we explored HCW views on acceptability and appropriateness of a novel strategy to increase human resources for health: placing youth interns as temporary LHWs in health facilities to support HIV services. HCWs found this to be a highly acceptable and appropriate strategy to strengthen HIV service delivery because the nature of the program was appealing, specifically its altruistic goals; implementation of the program was easy, once interns were placed at facilities; and the program yielded positive results. However, aspects of program implementation, namely the upfront burden posed by interns and limited communication with HCWs, detracted from the program’s overall acceptability and appropriateness. Overall, HCWs expressed strong affinity for YHA because they believed its benefits—to HCWs, patients, and interns—outweighed its costs.

Our results align with past research that found HCWs generally have favorable views of LHW programs that support HIV services [27], but the reason for the strong acceptance of the program was unique. The predominant finding in our study was that HCWs liked the YHA program because it was mutually beneficial. Similar to past research, HCWs appreciated the program because LHWs benefited the facility in important ways not captured by standard health metrics (e.g., reducing workloads and improving clinic functions) [11]. HCWs described overwhelmingly positive interactions with interns and did not describe worries about competition or incompetence found in other LHW programs [11, 26, 28, 40–42]. Most interestingly, YHA’s goal to empower youth, which is unique to this LHW program, appealed to HCWs’ sense of altruism, which strongly contributed to their favorable perception of the program. Altogether, these findings suggest that HCWs find the YHA program to be as acceptable and appropriate as other LHW programs, if not more so, precisely because of what makes YHA different from other LHW programs: its altruistic focus on empowering youth.

Despite the overwhelmingly positive reception of YHA, its implementation can be improved to maximize acceptability and appropriateness as it is scaled. Our findings emphasized that facility HCWs need to be included in planning for interns. This is echoed in past research, which shows HCWs are, unsurprisingly, happier with LHW programs when they have a voice in how the program is implemented, specifically with regards to how LHWs are introduced to facilities [27, 28]. Similarly, it is imperative that program implementers consider extending the length of the internship period to offset the upfront burden of training interns; otherwise, the costs of the program may eventually outweigh its perceived benefits.

### Policy implications

YHA’s appeal to HCW altruism through the program’s focus on youth empowerment may make this program attractive to policy makers striving to find new ways to strengthen human resources for health. Altruism is a key driver of HCW happiness and motivation, which has potential public health impact, as it can lead to reduced HCW attrition [9, 10]. To support HCW retention, researchers have proposed creating opportunities for HCWs to mentor others, as mentoring can improve morale by appealing to HCW altruism [10]. Our study suggests YHA could be such an opportunity, as it appears to improve HCW morale by enabling HCWs to engage in altruistic mentoring, while providing extra hands to reduce workloads, and thus lead to better workplace environment [8].

Moreover, the program’s multisectoral nature could make it attractive to policy makers as it could open new funding options for LHW programs. YHA has been funded through B-BBEE contributions, an economic empowerment policy [30]. The economic development sector, such as the African Development Bank, supports youth employment projects and could thus be a potential funder [43, 44]. The South African government has expressed interest in YHA because of its youth employment focus, while YHA’s focus on health system strengthening and HIV could also make it of interest to traditional public health entities (e.g., Department of Health and PEPFAR). A stakeholder analysis could be useful to elucidate their perspectives.

### Limitations

Although this mixed methods study provides a robust assessment of HCW perceptions of YHA, it was subject to several limitations. Firstly, we could only access HCWs working at facilities associated with Arum Institute and did not include facilities supported by other implementers of YHA; this limits the generalizability of our results, but we believe key drivers of acceptability and appropriateness would remain similar across facilities. Secondly, the validity of our survey questions is uncertain in our context, as key questions were validated in LMICs other than South Africa, and their original use was for mental health programs [36]. Thirdly, our interviewers were employees of Aurum, which helped implement YHA. This affiliation may have led to social desirability bias, with feedback perceived as impacting future Aurum support. However, similarity in findings between surveys and interviews suggest challenges with survey validity and social desirability bias were minimal. Fourthly, our study was limited to HCWs and did not address the experience of interns or patients. Finally, while our research highlights how HCWs perceive YHA to impact health facilities, we did not objectively measure such impact (e.g., morale, patient wait time). Nonetheless, this study is important as it provides a robust assessment of how HCWs perceive YHA when implemented under routine conditions.

## Conclusion

HCWs perceived having youth interns as temporary LHWs in facilities to be an acceptable and appropriate strategy to support HIV services in South Africa. While aspects of YHA implementation could be improved, HCWs found the program overwhelmingly acceptable and appropriate because its goals resonated with them, and they believed the program benefitted HCWs, patients, and interns. In particular, HCWs appreciated YHA’s altruistic nature, finding satisfaction in mentoring youth, suggesting this may be an especially effective LHW approach to strengthen human resources for health. Moving forward, we recommend adjustments to YHA’s implementation strategy to reduce burden on HCWs and research to quantify the program’s total cost and impact on patients and interns.

## Supplementary Information

**Additional file 1: Supplement 1.** Description of interns and healthcare workers engaged in the YHA program.

**Additional file 2: Supplement 2.** Logistic regression results (survey analysis).

## Data Availability

The datasets from this study are available from the corresponding author on reasonable request.

## Abbreviations

CHC: Community Health Center
HCW: Healthcare worker
HIV: Human Immunodeficiency Virus
LHW: Lay health worker
LMIC: Low- and middle-income country
NW: North West province
PEPFAR: U.S. President’s Emergency Plan for AIDS Relief
TFA: Theoretical Framework of Acceptability
YHA: Youth Health Africa
