# Supplementary material for: “They are gaining experience; we are gaining extra hands”: a mixed methods study to assess healthcare worker perceptions of a novel strategy to strengthen human resources for HIV in South Africa
Source: BMC Health Serv Res. 2023 Jan 11;23:27. doi: 10.1186/s12913-022-09020-z (PMC9832700; doi:10.1186/s12913-022-09020-z)
Supplement: Supplementary file 1 — Additional file 1: Supplement 1. Description of interns and healthcare workers engaged in the YHA program. [file 12913_2022_9020_MOESM1_ESM.docx]

**Supplement 1. Description of interns and healthcare workers engaged in the Youth Health Africa (YHA) program.**

| **Position** | **Description** | **Role in YHA** | **Funder** | **Inclusion in Study** |
| --- | --- | --- | --- | --- |
| Interns | 18-34 years old with secondary education but no employment experience, assigned as either programmatic interns or administrative interns | Programmatic interns serve as:  -HIV testing and counselors  -Peer navigators  -Tracers  Administrative interns serve as:  -Data capturers  -File clerks  -Admin clerks | YHA | Not included |
| Intern Supervisors | Healthcare workers that provide supportive supervision to numerous HIV-related HCWs, often across multiple facilities | -Provide supportive supervision to YHA interns on an as-needed basis  -Provide monthly updates on YHA performance to YHA | PEPFAR* implementing partner (e.g., Aurum Institute) | Surveys |
| Facility Supervisors | Nurses that support management and leadership of a facility’s HIV clinic. These often include nurses designated as the “operational manager”, “facility-in-charge nurse”, or those serving such roles in a temporary capacity. | No formal role or connection to YHA. These individuals provide overall management and leadership to the HIV unit at the facility. Since interns are part of this facility, they assume general oversight of the interns, but they do not report to YHA. | Department of Health or PEPFAR (through the implementing partner) | Interviews |
| Co-worker | Healthcare workers at the facility who worked directly with the interns. These staff could be in clinical roles (such as nurses), non-clinical programmatic roles (such as HIV testers and counselors) or administrative roles (such as data capturers). | No formal role or connection to YHA. These individuals work alongside YHA interns, either in the same positions (e.g., a data capturer working with a data capturer intern) or in different, but collaborating, positions (e.g., a nurse working with an intern who is supporting tracing activities). | Department of Health or PEPFAR (through the implementing partner) | Interviews |

*PEPFAR: President’s Emergency Program for AIDS Relief
