# Supplementary material for: “They are gaining experience; we are gaining extra hands”: a mixed methods study to assess healthcare worker perceptions of a novel strategy to strengthen human resources for HIV in South Africa
Source: BMC Health Serv Res. 2023 Jan 11;23:27. doi: 10.1186/s12913-022-09020-z (PMC9832700; doi:10.1186/s12913-022-09020-z)
Supplement: Supplementary file 2 — Additional file 2: Supplement 2. Logistic regression results (survey analysis). [file 12913_2022_9020_MOESM2_ESM.docx]

## SUPPLEMENT 2: Bivariate logistic regression results

Bivariate logistic regression analyses examining associations between intern supervisor characteristics and low total acceptability & appropriateness scores. *Row percentages are presented to support interpretation***.**

| **Variable** | | | **Lowest Acc & App* (n=13)** | **Higher Acc or App  (n=22)** | **Unadjusted OR** | **95% CI (Low)** | **95% CI (High)** | **p-value** |
| --- | --- | --- | --- | --- | --- | --- | --- | --- |
| **1. Province** | | |  |  |  |  |  |  |
| Gauteng | | | 9 (33%) | 18 (67%) | REF |  |  |  |
| North West | | | 4 (12%) | 29 (88%) | 0.3 | 0.07 | 1.1 | 0.06 |
| **2. Gender** | | |  |  |  |  |  |  |
| Male | | | 5 (20%) | 20 (80%) | REF |  |  |  |
| Female | | | 8 (23%) | 27 (77%) | 1.2 | 0.3 | 4.4 | 0.8 |
| **3. Supervisor Position** | | |  |  |  |  |  |  |
| Data monitor | | | 2 (11%) | 16 (89%) | REF |  |  |  |
| Counseling supervisor | | | 3 (18%) | 14 (82%) | 1.7 | 0.2 | 13.2 | 0.6 |
| Retention supervisor | | | 5 (29%) | 12 (71%) | 3.3 | 0.5 | 22.4 | 0.2 |
| Other (includes nurses) | | | 3 (37.5%) | 5 (62.5%) | 4.8 | 0.6 | 42.1 | 0.2 |
| **4. Duration in Current Position** | | | |  |  |  |  |  |
| <1 year | | | 3 (33.3%) | 6 (66.7%) | REF |  |  |  |
| 1-2 years | | | 3 (14%) | 19 (86%) | 0.3 | 0.05 | 2.2 | 0.2 |
| 3 or more years | | | 7 (24%) | 22 (76%) | 0.6 | 0.1 | 3.5 | 0.6 |
| **5. Duration as Intern Supervisor** | | | |  |  |  |  |  |
| <6 months | | | 3 (18%) | 14 (82%) | REF |  |  |  |
| 6-12 months | | | 5 (31%) | 11 (69%) | 2.1 | 0.4 | 11.8 | 0.4 |
| 1 or more years | | | 5 (18.5%) | 22 (81.5%) | 1.1 | 0.2 | 5.6 | 0.9 |
| **6. Current no. Interns**** | | |  |  |  |  |  |  |
| 1-2 interns | | | 7 (25%) | 21 (75%) | REF |  |  |  |
| 3-4 interns | | | 1 (7%) | 13 (94%) | 0.23 | 0.02 | 2.4 | 0.2 |
| 5+ interns | | | 4 (27%) | 11 (73%) | 1.1 | 0.2 | 4.9 | 0.9 |
| **7. Current no. Facilities with Interns**** | | | |  |  |  |  |  |
| 1 facility | | | 5 (29.4%) | 12 (70.6%) | REF |  |  |  |
| 2 facilities | | | 2 (8.3%) | 22 (91.7%) | 0.2 | 0.03 | 1.4 | 0.1 |
| 3 or more facilities | | | 4 (25.0%) | 12 (75.0%) | 0.8 | 0.2 | 4.0 | 0.8 |
| **8. Intern Role** | | |  |  |  |  |  |  |
| Program, no admin | | | 9 (30%) | 21 (70%) | REF |  |  |  |
| Admin, no program | | | 2 (10% | 17 (90%) | 0.27 | 0.04 | 1.6 | 0.2 |
| Admin & program interns | | | 1 (11%) | 8 (89%) | 0.29 | 0.03 | 3.1 | 0.3 |
| Other | | | 1 (50%) | 1 (50%) | 2.33 | 0.11 | 49.1 | 0.6 |
|  |  |  |  |  |  |  |  |  |

*Total score for acceptability and total score for appropriateness were ≤50, i.e., scores in the lowest quartile. **3 responses missing.

NOTE: Lower scores were uncommon, which is why we designed analysis to look at lower scores. It was not possible to differentiate extraordinarily high scorers, as most respondents had very high scores. We wanted to define "low" scores by having <= average score of 3 (moderate), but there were too few cases to examine. We did not run a multivariate regression analysis, as there were no significant findings in the unadjusted analysis.
